# Targeted Delivery of Sunitinib by MUC-1 Aptamer-Capped Magnetic Mesoporous Silica Nanoparticles

**DOI:** 10.3390/molecules28010411

**Published:** 2023-01-03

**Authors:** Mitra Torabi, Ayuob Aghanejad, Pouria Savadi, Abolfazl Barzegari, Yadollah Omidi, Jaleh Barar

**Affiliations:** 1 Research Center for Pharmaceutical Nanotechnology, Biomedicine Institute, Tabriz University of Medical Sciences, Tabriz 51656-65811, Iran; 2 Department of Pharmaceutics, Faculty of Pharmacy, Tabriz University of Medical Sciences, Tabriz 516664-14766, Iran; 3 Department of Environmental, Biological and Pharmaceutical Sciences and Technologies (Di.S.T.A.Bi.F.), University of Campania “Luigi Vanvitelli”, Via Vivaldi 43, 81100 Caserta, Italy; 4 Department of Pharmaceutical Sciences, College of Pharmacy, Nova Southeastern University, Fort Lauderdale, FL 33328, USA

**Keywords:** sunitinib, ovarian cancer, targeted drug delivery, anti-mucin 1 aptamer, magnetic mesoporous silica nanoparticles

## Abstract

Magnetic mesoporous silica nanoparticles (MMSNPs) are being widely investigated as multifunctional novel drug delivery systems (DDSs) and play an important role in targeted therapy. Here, magnetic cores were synthesized using the thermal decomposition method. Further, to improve the biocompatibility and pharmacokinetic behavior, mesoporous silica was synthesized using the sol-gel process to coat the magnetic cores. Subsequently, sunitinib (SUN) was loaded into the MMSNPs, and the particles were armed with amine-modified mucin 1 (MUC-1) aptamers. The MMSNPs were characterized using FT-IR, TEM, SEM, electrophoresis gel, DLS, and EDX. MTT assay, flow cytometry analysis, ROS assessment, and mitochondrial membrane potential analysis evaluated the nanoparticles’ biological impacts. The physicochemical analysis revealed that the engineered MMSNPs have a smooth surface and spherical shape with an average size of 97.6 nm. The biological in vitro analysis confirmed the highest impacts of the targeted MMSNPs in MUC-1 overexpressing cells (OVCAR-3) compared to the MUC-1 negative MDA-MB-231 cells. In conclusion, the synthesized MMSNP-SUN-MUC-1 nanosystem serves as a unique multifunctional targeted delivery system to combat the MUC-1 overexpressing ovarian cancer cells.

## 1. Introduction

Ovarian cancer is the fifth highest cause of tumor-related death among women worldwide, with a five-year survival rate of 35% [1]. Several approaches such as aggressive surgery, radiation, chemotherapy, and their combination are used for the treatment of ovarian cancer. Nonetheless, such treatment can be highly challenging considering the late diagnosis, existence of various subtypes, multi-drug-resistant nature of cancer, extensive side effects of antineoplastic agents, metastasis, and high recurrence rate after treatment. Moreover, some ovarian cancer patients whose cancer is recurrent or resistant to standard regimens are mostly incurable. Hence, the advent of early diagnostic tools and effective targeted therapy could be a promising approach to combat ovarian cancer [2].

Several studies have demonstrated that angiogenesis plays a crucial role in metastasis and the progression of ovarian cancer [3]. More than 12 different proteins are known to regulate the process of angiogenesis; among them, the vascular endothelial growth factor (VEGF) family and their receptors (VEGF-R) are considered critically important. It has clearly been shown that the VEGF and VEGFR expression is higher in ovarian epithelial carcinomas as compared to normal tissues. Therefore, targeting these markers could be a promising cancer treatment strategy [4]. Tumor-associated angiogenesis can be targeted using various medications such as SUN. SUN inhibits angiogenesis through the modulation of receptor tyrosine kinases (RTKs) pathways, including VEGFR1, VEGFR3, and platelet-derived growth factor receptors (PDGFR) [5]. About 80% of patients diagnosed with ovarian cancer are usually afflicted by the recurrent and drug-resistant type of ovarian cancer. Surprisingly, there is a significantly higher sensitivity to tyrosine kinase inhibitors than standard platinum-based therapy at this stage [6]. SUN has been used as a chemotherapy agent with moderate activity in some phase II studies of ovarian cancer research [7]; it seems that SUN could be added to the ovarian cancer treatment regimen in the future.

So far, due to off-target therapy and drug resistance, the effective treatment of ovarian cancer has not been achieved yet. Therefore, DDSs have become more meaningful. Because of the acidic pH of the tumor microenvironment (TME), induced by hypoxia and high cell metabolism, using pH-sensitive DDSs gets special consideration. Among the various functional DDSs, MMSNPs have become promising nanosystems for imaging and treatment applications due to their impressive properties [8,9].

The magnetic cores offer unique characteristics for efficient targeting, detection, and purification [10,11]. Several magnetic cores have been utilized for biomedical purposes, including magnesium oxide (MgO), cobalt oxide (Co_3_O_4_), nickel oxide (NiO), and iron oxide (Fe_3_O_4_). Among them, iron oxide is widely used for its potential in therapeutic and diagnostic applications due to its low toxicity, biocompatibility, and stability. However, the uncoated iron oxide tends to agglomerate, due to a high ratio of surface area to volume, making it prone to diagnosis by immune system cells. Hence, to overcome such drawbacks, an appropriate coating seems to be a suitable approach [12,13,14].

It has been shown that coating the magnetic cores with mesoporous silica nanoparticles (MSNPs), forms a uniform outer surface protecting them from the biological milieu and improving their performance as biomedical and/or therapeutic agents. MSNPs offer some unique characteristics: high loading capacity as a drug vehicle, high surface area, tunable pore diameters, and biocompatibility with non-toxic nature [15,16,17]. MSNPs can be easily modified with different ligands consisting of aptamers and antibodies for imaging and treatment application; furthermore, the special functional groups of MSNPs make it easy to control drug release [18,19]. Hence, the combination of the MSNPs with magnetic core facilitates the formation of an efficient carrier. The capabilities of the MMSNPs enable them to accumulate in the tumor tissue with passive targeting through increased permeability of tumor tissue blood vessels as well as the low lymphatic drainage, the so-called enhanced permeability and retention (EPR) effect [20]. Further, the conjugation of targeting agents on the surface of the MMSNPs enhances the accumulation in tumor tissue, so arming biomarkers to MMSNPs provides a valuable tool for the fabrication of the DDSs for active and passive targeting [21].

Of note, several potential biomarkers show a significant overexpression in ovarian cancers, such as MUC-1 glycoprotein [22]. MUC-1 is a highly glycosylated protein encoded by the mucin 1 gene that has been established for diagnostic applications, which is over-expressed in the most malignant epithelial cell surface; further, it has been chosen for early detection and treatment of cancers [23]. More than 90% of late-stage epithelial ovarian tumors and associated metastatic lesions exhibit overexpression of aberrant forms of MUC-1 [17]. The conjugation of targeting agents with MMSNPs enables them to target the tumor cells specifically. The single-stranded RNA or DNA oligonucleotides, so-called aptamers can detect and bind to the target molecules the same as the antibodies but with less immunogenicity and sensitivity. To the best of our knowledge, there is no previous report on using SUN-loaded MMSNPs, that are decorated with bioactive aptamers against MUC-1 in ovarian tumors. We propose that targeting ovarian cancer cells, using SUN-loaded MMSNPs and conjugated to MUC-1 aptamer, would improve its anti-cancer effect. It is assumed that the targeting agent (MUC-1 aptamer), results in the enhanced MMSNP-SUN-MUC-1 accumulation in the MUC-1 expressing cell line (OVCAR-3), as compared to MDA-MB-231 (as negative cell line). Consequently, such aptamer-directed targeting of late-stage ovarian cancer cells, may promote the uptake of the anti-cancer drug, overcome drug resistance, and limit the undesired effects of chemotherapy on healthy organs [24]. In this study, we engineered MMSNPs armed with the MUC-1 aptamer as a DDS for SUN. The biological impacts of nanocomposites (NCs) were evaluated on OVCAR-3 and MDA-MB-231 cells.

## 2. Results

**Figure 1:**
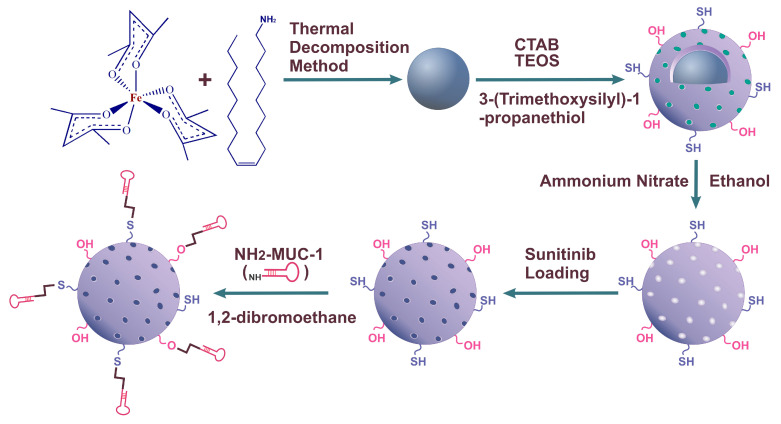
Schematic representation of the MMSNP-SUN-MUC-1 preparation. Magnetic cores were prepared using the thermal decomposition method and MMSNPs were fabricated using the sol-gel method, SUN was loaded into the MMSNPs, and NCs were grafted with MUC-1 aptamers.

According to Figure 1, the magnetic core was synthesized using the thermal decomposition method; immediately, they were coated with mesoporous structure using the sol-gel method. Then, MMSNPs were loaded with SUN and armed with amine-labeled MUC-1 aptamer. 

### 2.1. Fourier-Transform Infrared Spectroscopy (FT-IR) Spectroscopy

**Figure 2:**
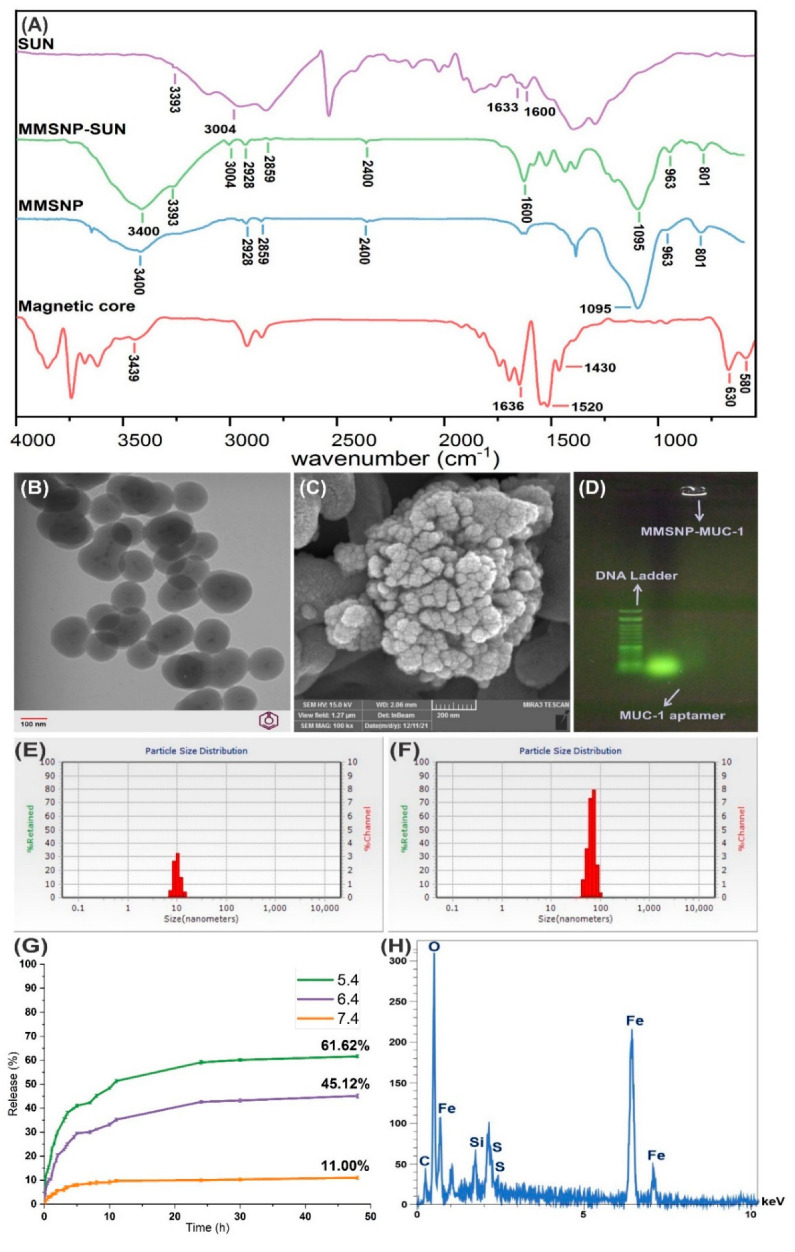
Physicochemical characterization of NCs; FT−IR spectra of the magnetic core, MMSNP, MMSNP−SUN, and SUN (**A**). TEM image and SEM image of the MMSNP (**B**,**C**). The gel electrophoresis analysis of aptamer armed MMSNP; The size of the DNA ladder applied in gel electrophoresis was 50 bp (**D**). The size distribution of the magnetic core and MMSNP−SUN, respectively (**E**,**F**). SUN release profile from MMSNP−SUN in phosphate buffer with different pH values (i.e., 7.4, 6.4 and 5.4) at 37 °C during 48 h (**G**). The EDX spectrum of the MMSNPs (**H**). The scale bar of TEM represents 100 nm and the scale bar of SEM represents 200 nm.

In the FT-IR spectra of the magnetic core, the absorption bands at 580 and 630 cm^−1^ are reported for stretching vibration mode to Fe-O [25,26]. The broad absorption band at 3439 cm^−1^ and a strong and sharp peak at 1636 cm^−1^ are attributable to O-H bond stretching and bending vibration of adsorbed water on the surface of the magnetic core [25,26,27,28]. Moreover, a strong peak at 1520 cm^−1^ and a peak at 1430 cm^−1^ are reported for the COO-Fe bond, which may relate to the reaction of hydroxide radical groups on the surface of Fe_3_O_4_ with carboxylate anion [29]. Further, the absorption band at 3400 cm^−1^ is related to the O-H bond stretching of water [30]. The most characteristic bands in the FT-IR spectra of MMSNP can be listed as absorption bands at 963 and 801 cm^−1^ associated with the symmetrical and asymmetrical stretching bond of siloxane and a strong band at 1095 cm^−1^, which shows the symmetrical stretching band of Si-O-Si [31]. The absorption stretching bond of the O-H related to water is at 3400 cm^−1^ [32]. The stretching band at 2400 is attributed to the SH bonds of MMSNPs [33]. In the sunitinib malate spectra, the absorption band at 1633 cm^−1^ indicates the amine functionalities of SUN, and the peaks at 1600 cm^−1^, 3393 cm^−1^, and 3004 cm^−1^ indicate the C=C stretching of the aromatic ring and amine group of SUN, respectively [34,35]. Further, the MMSNP-SUN spectra are similar to the MMSNP; except for the peaks at 1600 cm^−1^, 3393 cm^−1,^ and 3004 cm^−1^, which are related to SUN representing SUN’s successful load (Figure 2A) [35].

### 2.2. Morphological Studies

The transmission electron microscopy (TEM) and scanning electron microscopy (SEM) images of the MMSNPs, as presented in Figure 2B,C, revealed a monodisperse view with moderate uniformity, smooth surface, and spherical shape.

### 2.3. Agarose Gel Electrophoresis

The conjugation of MUC-1 aptamer to MMSNPs was verified by 1% agarose gel electrophoresis and UV spectroscopy. As illustrated in Figure 2D, the naked MUC-1 aptamer accelerated in the well compared to MMSNP-MUC-1 and showed a distinct band. MMSNP-MUC-1 did not show any distinct band.

### 2.4. Particle Size and Charge

The magnetic core and MMSNP size were about 10.9 nm and 97.6 nm, respectively with the charge of +15 mv and −10 mv and narrow polydispersity index (PDI) of 0.09 and 0.1 in the same order as presented in Figure 2E,F. The dynamic light scattering (DLS) measurement confirmed the nano-sized homogenous particles. The different sizes of the NCs in TEM and SEM with DLS may be due to dehydration during the imaging process [36].

### 2.5. Loading Efficiency and SUN Release Profile

The loading efficiency of SUN was 90 ± 0.36%. The SUN release from MMSNP-SUN was found to be in a pH-dependent manner. The mean cumulative release for 3 different pH values after reaching a steady state (48 h) indicated that the pH reduction from 7.4 to 5.4 increases SUN release. Figure 2G indicates that the cumulative percentage release at pH of 7.4, 6.4, 5.4 were 9.99 ± 0.47%, 42.65 ± 0.57%, and 59.12 ± 0.76% after 24 h and 11.00 ± 0.6%, 45.12 ± 0.84% and 61.62 ± 0.6% after 48 h, respectively.

### 2.6. Energy Dispersive X-ray (EDX) Analysis

The energy-dispersive X-ray spectroscopy of MMSNPs confirmed the presence of all constituting elements, including Si, Fe, C, O, and S atoms, as presented in Figure 2H.

### 2.7. Cell Cytotoxicity Assay

**Figure 3:**
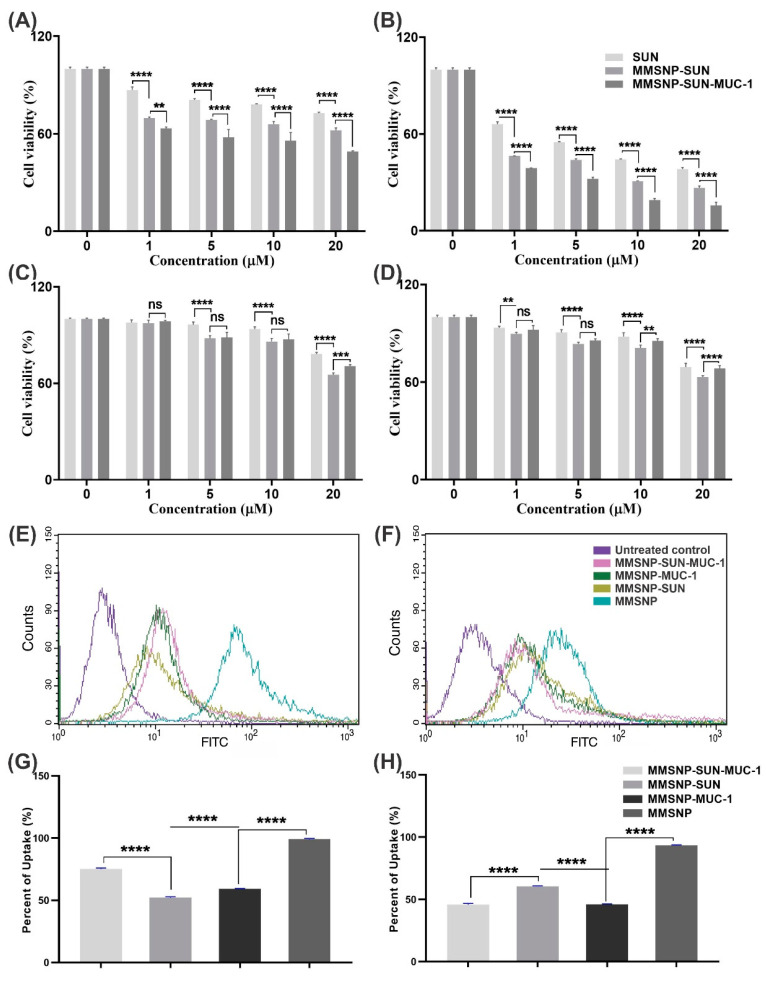
Dose- and time-dependent cytotoxicity of MMSNP-SUN-MUC-1, MMSNP-SUN, and SUN in OVCAR-3 cells and MDA-MB-231 cells, respectively after 24 h (**A**,**C**) and 48 h (**B**,**D**). The flow cytometry analysis of MMSNP-SUN-MUC-1, MMSNP-SUN, MMSNP-MUC-1, and MMSNP uptake in OVCAR-3 cells and MDA-MB-231 cells, sequentially (**E**,**F**). Quantification and comparison of NCs internalization into OVCAR-3 cells and MDA-MB-231 cells based on M2-mean fluorescent intensity, respectively (**G**,**H**). Data are shown as mean ± SD. The asterisks represent the following level of significance; not significant (ns.) *p* ≥ 0.05, ** *p* ≤ 0.01, *** *p* ≤ 0.001, **** *p* ≤ 0.0001.

In vitro cytotoxicity of the NCs was evaluated by the thiazolyl blue tetrazolium bromide (MTT) colorimetric assay in both cell lines. The cell viability of OVCAR-3 cells after 24 h of exposure to MMSNP-SUN-MUC-1, MMSNP-SUN, and SUN (at the concentration of 20 μM of SUN) was 49.1 ± 0.14%, 62.12 ± 0.18%, and 72.74 ± 0.31%, respectively, and after 48 h, the viability declined to 15.67 ± 0.26%, 26.65 ± 0.51%, and 38.37 ± 0.11%, with the *p*-value of <0.0001. The MDA-MB-231 cells’ viability after 24 h of exposure to the treatments was 70.63 ± 0.12%, 65.24 ± 0.16%, and 78.18 ± 0.08%. After 48 h declined to 68.40 ± 0.15%, 63.04 ± 0.06%, and 69.24 ± 0.09% as the same treatment order and *p*-value. These results confirmed that in OVCAR-3 cells, there was greater toxicity of MMSNP-SUN-MUC-1 than MMSNP-SUN due to MUC-1 overexpression. In contrast, in MDA-MB-231 cells, there was more significant toxicity of MMSNP-SUN than MMSNP-SUN-MUC-1. Generally, there was higher toxicity of SUN-loaded MMSNPs than SUN in both cell lines. Figure 3A–D displays the MTT assay results.

### 2.8. Cellular Uptake Study

The MUC-1 functionalized MMSNP-SUN was discovered for targeted therapy; hence to evaluate the targeted intracellular delivery, in vitro cellular uptake study was performed. As illustrated in Figure 3E,F, cells were exposed to fluorescein isothiocyanate (FITC)-labeled MMSNP-SUN-MUC-1, MMSNP-SUN, MMSNP-MUC-1, and MMSNP for 2 h (in the equal concentration of 20 µM SUN). It was anticipated that the uptake of MMSNP-SUN-MUC-1 in OVCAR-3 cells was significantly higher than MMSNP-SUN (Figure 3E). Moreover, in MDA-MB-231 cells, there was a greater internalization of MMSNP-SUN than MMSNP-SUN-MUC-1 as shown in Figure 3F, which can be interpreted by steric hindrance of MUC-1 limiting the correlation of MMSNPs with the cell surface. Figure 3G,H indicates the uptake data histogram in both cell lines.

### 2.9. Quantification of Apoptosis/Necrosis Assessment

**Figure 4:**
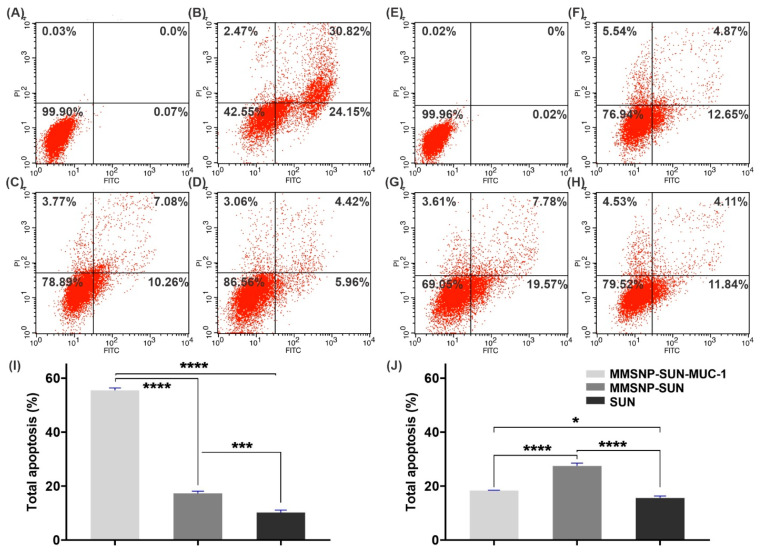
Apoptosis and necrosis evaluation. Annexin V/FITC and PI flow cytometric detection of apoptosis and necrosis in OVCAR-3 cells and MDA-MB-231 cells, respectively treated with normal media as control (**A**,**E**), MMSNP-SUN-MUC-1 (**B**,**F**), MMSNP-SUN (**C**,**G**) SUN (**D**,**H**) after 24 h. The histogram of total apoptosis data in OVCAR-3 cells and MDA-MB-231 cells sequentially (**I**,**J**). Data are shown as mean ± SD. The asterisks represent the following level of significance; not significant * *p* ≤ 0.05, *** *p* ≤ 0.001, **** *p* ≤ 0.0001.

For detecting the cell surface phosphatidylserine (PS) and DNA damage, the cells were stained with annexin V/FITC and propidium iodide (PI). Figure 4 shows the apoptosis/necrosis rate in OVCAR-3 and MDA-MB-231 cells with various treatments after 24 h. The MMSNP-SUN-MUC-1 treated OVCAR-3 cells showed a significantly higher apoptosis rate in comparison to MMSNP-SUN and SUN. Furthermore, in MDA-MB-231 cells apoptosis rate of MMSNP-SUN was higher compared with MMSNP-SUN-MUC-1 and SUN. These findings mentioned a higher incidence of apoptosis in both cell lines than necrosis which indicates apoptosis is the primary mechanism of cell death. Moreover, Figure 4I,J indicates the histogram analysis of apoptosis in both cell lines.

### 2.10. Gene Expression Analysis

**Figure 5:**
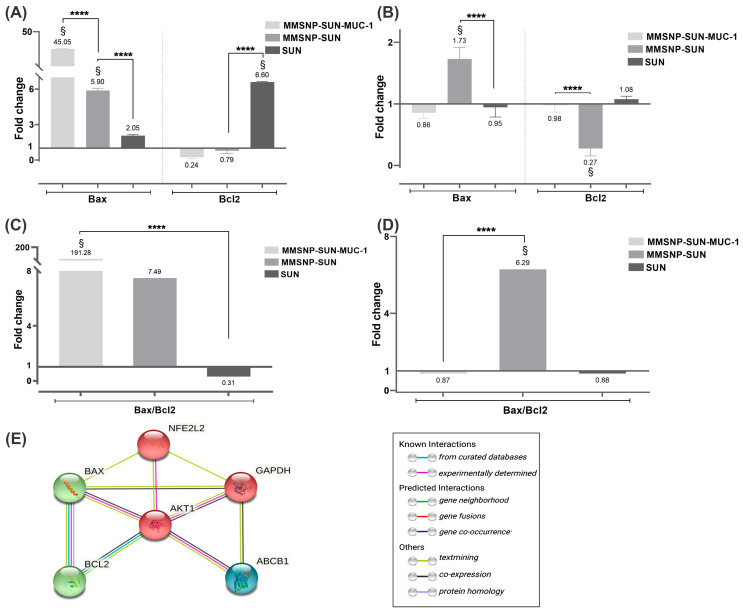
The gene expression analysis by real-time PCR; Bax and Bcl2 (**A**,**B**) and the ratio of Bax/Bcl2 (**C**,**D**), respectively in OVCAR-3 cells and MDA-MB-231 cells. The protein–protein interactions (PPI) of apoptosis-related proteins and antioxidant enzymes analysis using STRING v. 10.5 software with a medium confidence score of 0.400 and Kmeans clustering algorithm with 3 clusters (**E**). §: Statistical significance (*p* < 0.05) compared with untreated control (Y = 0). The asterisks represent the following level of significance; not significant **** *p* ≤ 0.0001.

The mRNA expression of Bax, Bcl2 genes, and the Bax/Bcl2 ratio were evaluated in OVCAR-3 and MDA-MB-231 cells. The expression values were normalized based on the expression of GAPDH as the housekeeping gene. As presented in Figure 5A,B, treating OVCAR-3 cells with SUN, MMSNP-SUN-MUC-1 and MMSNP-SUN increased the expression of the Bax (mostly in MMSNP-SUN-MUC-1 treated cells). However, in MDA-MB-231 cells, there were no significant expression changes in the expression of genes except in MMSNP-SUN treated cells. Further, the Bcl2 expression level was generally reduced in OVCAR-3 cells, especially in MMSNP-SUN-MUC-1 treated; and MMSNP-SUN treated MDA-MB-231 cells, while the changes in other treatments was not significantly different than the un-treated control.

As presented in Figure 5C,D, the ratio of Bax/Bcl2 is significantly higher in OVCAR-3 cells treated with MMSNP-SUN and MMSNP-SUN-MUC1 as compared to control, while in MDA-MB-231 cells this ratio is higher only in MMSNP-SUN treated samples.

### 2.11. In Silico Protein–Protein Interaction

Figure 5E indicates the association of apoptosis-related proteins and antioxidant enzymes in oxidative stress conditions. This network showed that most of these target genes interact with each other, indicating that the apoptosis maybe is mediated through oxidative stress.

### 2.12. DAPI Staining

**Figure 6:**
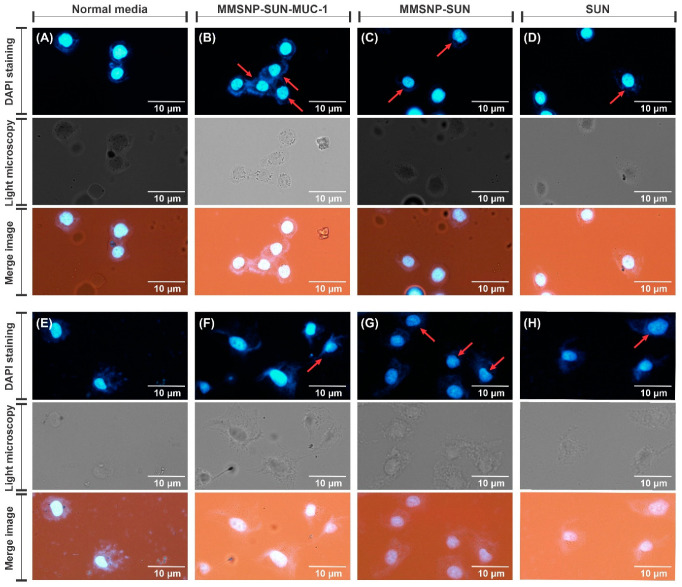
The nuclear morphological change analysis (DAPI staining image, light microscopy, merge images) of OVCAR-3 cells and MDA-MB-231 cells, respectively after exposure to normal media (**A**,**E**), MMSNP-SUN-MUC-1 (**B**,**F**), MSNP-SUN (**C**,**G**) and SUN (**D**,**H**).

The induction of apoptosis by MUC-1 targeted and untargeted MMSNP-SUN in both cell lines was studied using microscopic analysis of DAPI-stained cells. The morphological changes of the nucleus and chromatin condensation are the main hallmarks of apoptosis. Figure 6 shows the fluorescent and light microscopy images of the OVCAR-3 and MDA-MB-231 DAPI stained cells. The morphological changes with fragmented and condensed chromatin were more detectable in MMSNP-SUN-MUC-1 than in other treatments in the OVCAR-3 cell line and MMSNP-SUN in the MDA-MB-231 cell line after 24 h. In MMSNP-SUN-MUC-1 treated OVCAR-3 cells, the nucleus was brighter with disintegrated parts, demonstrating apoptosis induction.

### 2.13. Intracellular Reactive Oxygen Species (ROS) Assay

**Figure 7:**
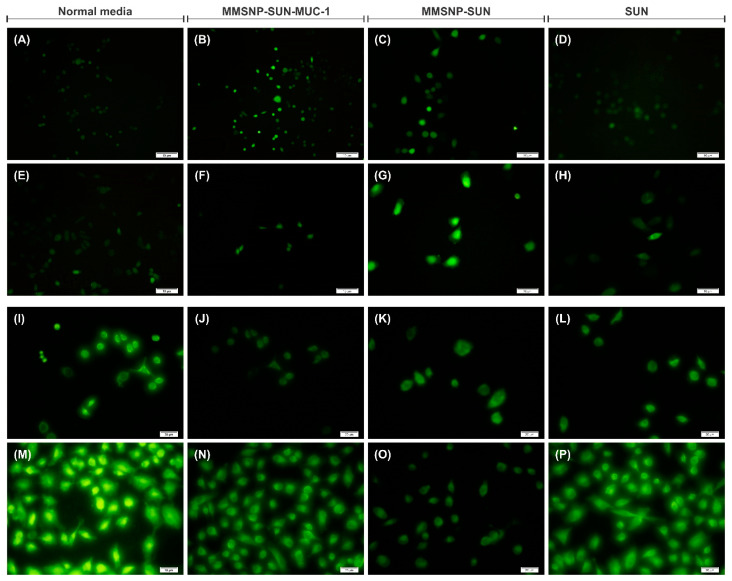
ROS production analysis on OVCAR-3 and MDA-MB-231 cells; sequentially treated with normal media as control (**A**,**E**), MMSNP-SUN-MUC-1 (**B**,**F**), MSNP-SUN (**C**,**G**) and SUN (**D**,**H**). Rhodamine 123 staining for the mitochondrial membrane potential analysis in OVCAR-3 and MDA-MB-231 cells; respectively treated with normal media as control (**I**,**M**), MMSNP-SUN-MUC-1 (**J**,**N**), MSNP-SUN (**K**,**O**) and SUN (**L**,**P**). The scale bar of images represents 20 μm.

To evaluate the effect of naked-/MUC-1-MMSNP-SUN on intracellular ROS production, DCHF-DA fluorescent dye was used. As shown in Figure 7A–H, increasing the fluorescent signal intensity of MMSNP-SUN-MUC-1 treated OVCAR-3 (Figure 7B), and MMSNP-SUN treated MDA-MB-231 (Figure 7G) reveals the maximum ROS production. In addition, SUN treatment has a slightly low effect on ROS production in both cell lines.

### 2.14. Mitochondrial Membrane Potential Analysis

As presented in Figure 7I–P, the untreated control cells showed the highest fluorescence, indicating the polarized mitochondrial membrane in these cells (Figure 7I,M). Moreover, the treatments blocked the energy metabolism and induced apoptosis in MMSNP-SUN-MUC-1 treated OVCAR-3 (Figure 7J) and MMSNP-SUN treated MDA-MB-231 (Figure 7N).

## 3. Discussion

MMSNPs are widely used as site-specific DDSs to maximize the expected therapeutic effect and also to reduce the undesired adverse effects of chemotherapeutic agents such as SUN [37]. Several clinical studies demonstrated its effectiveness in some ovarian cancer clinical studies [38,39]; however, its moderate efficacy has limited its widespread use in the clinical setting. Therefore, in the present study, to improve the effectiveness of SUN, MMSNP armed with MUC-1 aptamer was used for active targeted drug delivery to the ovarian cancer cells. The use of aptamers as targeting agents has attracted much attention due to their unique characteristics. They are considered a suitable alternative to antibodies since the huge ligand conjugation changes the size and pharmacokinetics of the nanoparticles; whilst using aptamers has minimal impact on the size of nanoparticles due to their small size [40]. Furthermore, according to the binding-site barrier phenomenon, it seems that aptamers are a better choice for deep and uniform penetration of the delivery system inside the tumor tissue [40,41].

Dispersibility in liquids is crucial for a suitable nano-sized delivery system. Therefore, the mesoporous silica coating increases the dispersity of the nanosystem and prevents the loss of magnetism of the magnetic core by preventing aggregation [42]. On the other hand, MMSNPs can improve the solubility of poorly soluble drugs like SUN [43]. The MSNP coating also masks the positive charge of the magnetic core, making them less toxic to the non-cancerous cells [42].

For covalent conjugation of the MUC-1 aptamer to NCs, a linker is required. Thiolation of these NCs not only facilitates the binding of desired functional groups but also, thiolation slows down the aggregation of the particles [44]. In the current study, the magnetic cores were synthesized using the thermal decomposition method. Then, cores were surrounded by thiol-modified mesoporous silica nanoparticles using the sol-gel method, and then the preparation of the delivery system was continued by SUN loading and MUC-1 aptamer conjugation.

The physicochemical characterizations were performed during each step of the preparation. Accordingly, the FT-IR spectra confirmed the essential characteristic bands related to the magnetic core, MMSNP, MMSNP-SUN, and SUN and further validated the successful synthesis process (Figure 2A) [25,29,31,35]. Figure 2B,C indicated the formation of the mesoporous silica coat on spherical Fe_3_O_4_ nanoparticles and the surface morphology of the MMSNPs using the TEM and SEM imaging process. As presented in Figure 2D, the conjugation of MUC-1 aptamer to MMSNP was confirmed as visualized by agarose gel electrophoresis. MMSNP-MUC-1 showed a retarded band in agarose gel; similar to the observation of Jafari et al., which confirmed the conjugation of MUC-1 to the surface of nanoparticles [45]. Besides, according to Xie et al., to evaluate the conjugation of EpCAM aptamer to MSNPs, polyacrylamide gel electrophoresis was carried out with a similar result [46].

As stated, our synthesized magnetic core and MMSNPs with average size of 10.9 nm and 97.6 nm (Figure 2E,F) are in the optimal size range required for passive tumor targeting by the EPR effect [47,48]. Therefore, aptamer-armed MMSNP-SUN can accumulate in tumor tissue due to the passive mechanism, and the specific targeting aids to maximize uptake by the targeted cells [43].

The cumulative drug release results from MMSNPs showed an efficiently pH-responsive behavior at 37 °C and made the present MMSNPs promising candidates for anticancer-targeted drug delivery (Figure 2G). The pH-dependent manner of release was owing to the presence of tertiary amine groups in the SUN chemical structure (pKa = 9.8) and its electrostatic adsorption on the surface of the MMSNPs. At the lower pH values, protonated amino groups of loaded SUN improve the solubility [49,50]. The low release at physiological pH before reaching the TME can remarkably hinder the undesired adverse effects [48]. In a previous study, we have shown that the release of doxorubicin can reach the highest level when it is placed in an acidic environment with a pH value similar to that of endosomal compartments [51]. According to Li and colleagues, the release of the synthesized MSNPs with the base-catalyzed co-condensation method was pH-dependent and was increased in the acidic environment [19].

The EDX spectrum of the MMSNPs showed the existence of Si, Fe, C, O, and S in the NCs (Figure 2H). Additionally, the punctual EDX analysis showed the Si and Fe element overlapping, which can validate the successful MMSNPs synthesis process. According to Ruiz-Hernandez et al., the EDX spectrum of the MMSNPs synthesized by the co-precipitation method was similar to our nanoparticles [52].

The in vitro cytotoxicity of functionalized MMSNPs was studied on OVCAR-3 and MDA-MB-231 as the MUC-1 positive and negative cell lines, respectively (Figure 3A–D).

The IC50 of SUN was 8.47 μM in OVCAR-3 cells, and 42.7 μM in MDA-MB-231 after 48 h. This clearly highlights the sensitivity of OVCAR-3 cells to this receptor tyrosine kinase inhibitor. SUN acts via various mechanisms, including PDGFR. Interestingly thrombopoietin (TPO) is considered as a biomarker for ovarian cancer, that also regulates expression of PDGFR [53]. Taken altogether, we can conclude that SUN is more effective in inhibition of OVCAR-3 cell growth as compared to MDA-MB-231 cells, that lacks TPO expression [54].

In OVCAR-3, the MMSNP-SUN-MUC-1 treated cells showed more toxicity than others, also in MDA-MB-231, MMSNP-SUN treated cells showed the most toxicity in the same incubation time (24 and 48 h). It should be stated that the toxicity of targeted and untargeted MMSNPs was not beyond 5% in both cell lines, which showed that they were nontoxic for both cell lines. Both cell lines’ viability was reduced while exposed to treatments in a dose-/time-dependent manner. According to Sheng et al., mucin 13 aptamer as the predicting biomarker in A-704, A489, ACHN, and HEK239 cells demonstrated a synergic effect with SUN, promising results in prognosis and resistance decrease. Accordingly, the MTT assay results showed higher toxicity in mucin 13 expressing cells [55]. Further, in a study reported by Savla, the MUC-1 doxorubicin conjugated quantum dots showed similar results in the MUC-1 expressing A2780/AD ovarian cancer cells [56]. Zhang et al. developed silicon nano-dot-based aptasensors and showed high selectivity and toxicity in the MUC-1 overexpressed cells; the MCF-7 cells were the positive cell line and MCF-10A and Vero cells were as negative cell lines [57].

The FITC-labeled flow cytometry assay results attributed to the higher uptake level of MMSNP-SUN-MUC-1 compared to MMSNP-SUN in OVCAR-3 cells and lower uptake level of MMSNP-SUN-MUC-1 compared with MMSNP-SUN in MDA-MB-231 cells (Figure 3E,F). It can be presumed that through the specific interaction of MUC-1 aptamer with its cellular membrane receptor, the targeted MMSNPs actively accumulate inside the positive cells, showing higher cellular internalization. The interaction of negatively charged MUC-1-armed MMSNPs with the like charge surface of cells leads to lower uptake levels in negative cell lines [58]. The aptamer’s spatial hindrance [59] limited the MMSNP-SUN-MUC-1 and MMSNP-MUC-1 uptake in negative cell lines. According to the histogram analysis, as presented in Figure 3G,H, the low spatial hindrance of MMSNP can justify the highest uptake (98% in OVCAR-3 cells and 93% in MDA-MB-231 cells). The uptake of MMSNP-SUN-MUC-1 and MMSNP-SUN in OVCAR-3 cells (74.64% and 51.7%, respectively) indicated that conjugation with MUC-1 aptamer led to the selective uptake of the MMSNPs in positive cell lines. MUC-1 conjugated MMSNPs synthesized by Hanafi Bojd et al. exhibited fairly similar uptake results in MCF-7 and CHO cells as our targeted nanoparticles [60]. According to Xie et al., the results of the uptake study of the doxorubicin-loaded EpCAM-armed MSNPs were the same way as our uptake study [46].

Annexin V/FITC and PI assay was performed to demonstrate the early/late apoptosis (Figure 4) in the cells upon treatment with MMSNPs. The total apoptosis rates of MMSNP-SUN-MUC-1 and MMSNP-SUN in OVCAR-3 cells (55.49% and 17.33%, respectively) showed that SUN treatment, especially when loaded in the MUC-1 targeted system results in higher apoptotic cell death. In early apoptosis, the PS is translocated to the outer membrane to trigger phagocytes [61]. According to Ping and colleagues, SUN enhances the apoptosis by Bacillus Calmette-Guerin in T24 cells [62]. Additionally, Xie et al. reported that based on the annexin V/FITC and PI staining, a higher apoptosis rate was observed in doxorubicin-loaded aptamer-armed MSNPs in SW480 cells [46].

Gene expression analysis was carried out to further explore the effect of targeted and untargeted MMSNP-SUN treatment on cellular pathways in both cell lines. The changes in the mRNA levels of the genes between the untreated and treated cells were normalized using the GAPDH as the housekeeping gene by the Pfaffl method [63]. Figure 5 presents the real-time polymerase chain reaction (real-time PCR) results of Bax, Bcl2, genes. The analysis of Bax as a pro-apoptotic gene and Bcl2 as an anti-apoptotic gene showed that cell death may occur mainly by apoptosis. It was observed that there was an up-regulation of Bax and down-regulation of Bcl2 in MMSNP-SUN-MUC-1 treated OVCAR-3 cells. Moreover, there was a Bax up-regulation and a Bcl2 down-regulation in MMSNP-SUN-treated MDA-MB-231 cells. This clearly shows that the OVCAR-3 cells that are MUC-1 positive, have higher fold-change in the treatment with aptamer conjugated system, while in MDA-MB-231 cells, the aptamer conjugation rather create a spatial obstruction for drug transport, and the highest increase is observed in un-armed nanosystem. The increase in Bax/Bcl2 ratio shows that the prepared DDS can be an effective platform to combat ovarian cancer, in this case, the MUC-1 overexpressed cells are more prone to apoptosis through targeted MMSNPs. Moreover, the association of the pre-apoptotic genes was displayed in Figure 5E according to STRING v. 10.5 software. According to in silico results, the apoptosis mediated cell death might occur through up-regulation of Nrf2 expression and can be associated with ROS release; under oxidative stress conditions [64].

Moreover, the expression of drug-resistance genes has a critical role in apoptosis, that can be bypassed by nanoformulation. According to the statistics, drug resistance is common in ovarian cancer; hence, defeating drug resistance could be a great approach [65,66]. Furthermore, the Akt1 gene expression regulates cell metabolism, proliferation, and survival, that could potentially prevent cell apoptosis.

As illustrated by DAPI staining (Figure 6), the MMSNP-SUN-MUC-1 treated OVCAR-3 cells showed the clearest morphological changes. Additionally, condensation was more detectable in MMSNP-SUN-treated MDA-MB-231 cells. In a study by Wang et al., renal cell carcinoma cells treated with wogonin and SUN showed a similar pattern of DNA damage [67]. The microscopic evaluation in both cell lines further confirmed the results of other biological evaluations. As shown by Wang et al., the OVCAR-3 cells treated with saponins showed similar crescent-shaped nuclei [68] as are present in the cells treated with MMSNP-SUN-MUC-1. Likewise, the study by Afsar et al. showed similar morphological changes treated with *Acacia hydaspica* in MDA-MB-231 cells [69]. The ROS evaluation showed the oxidative stress level in the cells. As shown in Figure 7A–H, the remarkable ROS release was generated by MMSNP-SUN-MUC-1 in OVCAR-3 cells and MMSNP-SUN in MDA-MB-231 cells compared to other treatments. This imaging data was in agreement with gene expression data in the cells treated with the same sample. As shown in research by Wang et al., the OVCAR-3 cells treated with saponins showed a similar ROS release as our cells exposed to MMSNP-SUN-MUC-1 [68]. The mitochondrial membrane potential analysis was evaluated by rhodamine 123 staining. As presented in Figure 7I–P the untreated control cells in both cell lines showed the highest rhodamine 123 accumulation. In research by Irannejad et al., the doxorubicin-resistant MCF-7 cells showed the highest rhodamine 123 staining in untreated and metformin-treated cells [70].

## 4. Material and Methods

### 4.1. Materials

Cetyl trimethylammonium bromide (CTAB), tetraethyl orthosilicate (TEOS), sodium hydroxide (NaOH), ethyl acetate, 3-(Trimethoxysilyl)-1-propanethiol, ammonium nitrate (NH_4_NO_3_), dimethyl sulfoxide (DMSO), 1,2-dibromoethane, n-hexane, benzyl ether, iron (Ⅲ) acetylacetonate, and oleylamine were obtained from Sigma Aldrich (Dorset, UK). 2′,7′-dichlorodihyofluorescin diacetate (DCFH-DA), and MTT were purchased from Sigma Aldrich (Saint Louis, MO, USA). Fluorescein isothiocyanate (FITC)-labeled annexin-V/propidium iodide (PI) was obtained from ApoFlowEx FITC kit, EXBIO, (Prague, Czech Republic). TRIzol reagent was purchased from Invitrogen (Carlsbad, CA, USA). SYBR green master mix was obtained from Biofact (Daejeon, Republic of Korea). Human ovarian cancer OVCAR-3 and human breast cancer MDA-MB-231 cell lines were obtained from the National Cell Bank of Iran, Pasteur Institute (Tehran, Iran). Amine-modified MUC-1 aptamer with the sequence of (5′-NH_2_ (C6) GCAGTTGATCCTTTGGATACCCTGG-3′) [71] was purchased from Bioneer (Daejeon, Republic of Korea). All other media and cell culture components not listed were purchased from SPL life sciences (Geumgang, Republic of Korea) and Gibco (Paisley, UK).

### 4.2. Preparation of Magnetic Core

The magnetic core was prepared using thermal decomposition process as previously described with some modifications [51,72]. Briefly, the solution of iron (Ⅲ) acetylacetonate (0.22 g, 0.62 mmol) in 6 mL benzyl ether and oleylamine (1:1 *V*/*V*) was dehydrated for 2 h at 105 °C. Next, with the addition of ethanol (40 mL) the temperature of the reaction medium was raised to and kept at 300 °C for 3 h. The magnetic core was then collected by centrifugation at 7000× *g*, after which it was dispersed in n-hexane (10 mL) and kept at 4 °C.

### 4.3. Preparation of Thiol Functionalized MMSNP

The suspension of magnetic core (100 mg) in 100 µL chloroform was drop-wisely added to the solution of CTAB (125 mg, 0.34 mmol) in 30 mL of deionized water while stirring the mixture at the temperature of 60 °C. Then, the pH was adjusted to 12 with the addition of NaOH (0.2 N). Following, the solution of TEOS (1000 mg, 5 mmol) in ethyl acetate (1:4 *V*/*V*) was added to the reaction mixture while stirring at 70 °C for 2 h. Then, the solution of 3-(Trimethoxysilyl)-1-propanethiol (20 mg, 0.10 mmol) in ethyl acetate (1 mL) was added to the medium and stirred for 3 h at the same temperature; next, MMSNPs were centrifuged at 2800× *g*, separated and washed using ethanol two times (×2). Afterward, MMSNPs were re-dispersed in an ethanolic solution of ammonium nitrate (10 mg/mL) and stirred for 3 h at 60 °C. Next, the NCs were washed with ethanol (×3) and collected by centrifugation at 2800× *g*.

### 4.4. Drug Loading (MMSNP-SUN)

The SUN was physically incorporated in MMSNPs. To this end, the SUN (50 mg) in DMSO (2 mL) was added to the MMSNP (50 mg), and the resulting reaction mixture was stirred overnight at room temperature (RT). Afterward, the NCs were centrifuged at 5000× *g* and the supernatant was allowed to evaporate at 45 °C in the oven. Then, the solution of the leftover powder in 1 mL of DMSO was used to determine SUN loading efficiency. MMSNP-SUN was dried using the Labconco vacuum freeze-dryer (Kansas City, MO, USA).

### 4.5. SUN Loading Efficiency

The loading efficiency of SUN was evaluated with Ultraviolet/Visible (UV/Vis) spectrophotometry (Cecil CE 7500, Cambridge, UK) at λ max of 267 nm and calculated using the following equation:

SUN loading Efficiency = 100 × (Total SUN added—Unloaded SUN)/Total SUN added.

### 4.6. Conjugation of MUC-1 Aptamer to MMSNP-SUN (MMSNP-SUN-MUC-1)

The suspension of MMSNP-SUN (1.5 mg) in nuclease-free water (600 μL) was added to the solution of 5′–NH_2_–MUC-1 aptamer (2.5 nmol) in nuclease-free water (100 µL) and 1,2- dibromoethane (10 μL) and incubated under constant mild shaking overnight at RT. Next, the MUC-1 armed nanoparticles were collected using an external magnetic field, washed with PBS (×3), re-suspended in nuclease-free water, and kept at 4 °C. The schematic representation in Figure 1 illustrates the NCs synthesis process.

### 4.7. Conjugation of FITC to NCs

To evaluate the in vitro cellular uptake of the NCs by flow cytometry, MMSNPs were labeled with FITC. To this end, 0.5 mL of NCs suspension (7.8 mg/mL) in DMSO was mixed with the 0.5 mL solution of FITC in DMSO (2 mg/mL) and stirred overnight. Then, the final products were collected using centrifugation at 3100× *g* and washed with PBS (×3), and used for uptake flow cytometry studies.

### 4.8. Instrumentation

#### Characterization of the NCs

The surface modifications and the functional groups of the structures were verified using FT-IR spectroscopy (Bruker Optik GmbH, Ettlingen, Germany) in the range of 500–4000 cm^−1^. The morphology and size of MMSNPs were evaluated by typical TEM using Carl Zeiss, LEO 906E (Germany) microscope. The surface morphology of MMSNP was investigated by field emission scanning electron microscope (FESEM) using MIRA3 FEG-SEM (Tescan, Czechia). The ξ-potential and size of MMSNPs were measured by a DLS analyzer Zetasizer, Nanotrac wave (Microtrac Inc., Montgomeryville, PA, USA). Additionally, the PDI was evaluated to illustrate the size distribution of nanoparticles. The elemental composition of materials was established by EDX using MIRA3 FEG-SEM (Tescan, Czechia).

### 4.9. Agarose Gel Electrophoresis

To confirm the conjugation of MUC-1 aptamer to the NCs, the MMSNP, MMSNP-MUC-1 (1 mg), and free aptamer (3.2 nmol) were loaded into the agarose electrophoresis gel (1% *W*/*V*). The electrophoresis was performed at 70 V for 1 h, and the gel was visualized under UV illumination (Syngene IG/LHR-E, Cambridge, UK).

### 4.10. In Vitro Drug Release Study

For the assessment of drug release from SUN-loaded MMSNPs; MMSNP-SUN was suspended in 2 mL of phosphate buffer medium (2.5 mg/mL) with three different pH values as the condition of endosomes (pH = 5.4), TME (pH = 6.4), and blood (pH = 7.4). The suspensions were loaded into a pre-swollen dialysis membrane bag (cutoff 2 kDa, Sigma Aldrich). The dialysis bag was then immersed in 198 mL of release medium and incubated at 37 °C for 48 h. In predesignated time points, 2 mL of samples were withdrawn from the release medium and replaced with the equivalent amount of fresh medium, so the total volume of the solutions was kept constant. The SUN release from MMSNPs was estimated using UV/Vis spectrophotometry at 267 nm.

### 4.11. Cell Culture

The OVCAR-3 and MDA-MB-231 cells were cultured and incubated with RPMI-1640 with 10% fetal bovine serum (FBS), penicillin (100 IU/mL), and streptomycin (100 µg/mL) and kept at CO_2_ (5%) incubator at 37 °C with 98% humidity.

#### In Vitro Cell Viability Assay

The in vitro cell cytotoxicity of the SUN-loaded, MUC-1 targeted, and untargeted MMSNPs was investigated in the OVCAR-3 and MDA-MB-231 cells. Both cell lines were cultivated in 96-well plates with a seeding density of 3 × 10^3^ cells/well. After 24 h, the cells were incubated with varying concentrations of MMSNPs based on the equivalent concentration of SUN (1, 5, 10, and 20 µM) for 24 and 48 h. After the removal of media, cells were exposed to 20 μL of MTT solution (5 mg/mL) with 180 μL of the fresh media. After incubation for 4 h, the media was substituted with DMSO (200 µL) and Sorenson’s buffer (25 µL). The absorbance was measured at 570 nm using an Elisa plate reader (ELx808, BioTek Instruments, Winooski, VT, USA).

### 4.12. Flow Cytometry Analysis

#### 4.12.1. MMSNPs Uptake Evaluation

The flow cytometry analysis was performed to evaluate the in vitro cellular uptake of NCs. Briefly, both cell lines were cultivated in 6-well plates (9 × 10^4^ cells/well) for 24 h and treated with the FITC loaded, MUC-1 targeted, and untargeted MMSNPs allowing the internalization to occur for 2 h. The cells were then washed with PBS (×3), trypsinized, centrifuged at 160× *g*, and re-suspended in PBS. The samples were analyzed using the FACS Calibur^®^ flow cytometer (Becton Dickinson, San Jose, CA, USA), with a minimum number of 1.0 × 10^4^ cells/per sample.

#### 4.12.2. Assessment of Apoptosis/Necrosis

To measure the apoptosis/necrosis rate of cells treated with the engineered formulations, they were stained with FITC-labeled annexin-V/PI and analyzed using FACS Calibur^®^ flow cytometer (Becton Dickinson, San Jose, CA, USA). To this end, the cells were cultivated in 6-well plates (9 × 10^4^ cells/well) for 24 h and then treated with normal media, MMSNP-SUN-MUC-1, MMSNP-SUN, MMSNP-MUC-1, MMSNP, and SUN. The treated cells were detached after 24 h, washed, re-suspended in binding buffer (100 µL) and FITC-labeled annexin-V/PI (10 µL), and incubated in a dark for 20 min. Then, the cells were subjected to the FACS Calibur^®^ flow cytometer.

### 4.13. Gene Expression Analysis

The real-time PCR was conducted to investigate the expression of different genes in the treated cells. The real-time PCR reactions were performed for the apoptosis pathway (Bax and Bcl2, and their expression ratio). Both cell lines were cultivated in 6-well plates (9 × 10^4^ cells/well) for 24 h and then treated with engineered formulations. After 24 h, total RNAs were extracted using TRIzol reagent according to the previous instructions [73]. The RNA concentration and purity were determined using a Nano-Drop spectrophotometer (Thermo Fisher Scientific Inc., Wilmington, DE, USA). According to Thermo Fisher Scientific reverse transcription instructions, the RNA yielded to cDNA. Next, real-time PCR was performed. For the real-time PCR analysis, cDNA (1 μL), SYBR Green Master Mix (10 μL), nuclease-free water (8.8 μL), and forward and reverse primers (0.5 µL of each primer, 10 nM) were prepared and loaded on Bio-Rad iQ5 Real-time PCR system (Bio-Rad, Montreal, QC, Canada; FDA) and analyzed using iQ5 optical system software. GAPDH gene expression was applied for the normalization of mRNA expression levels. All the samples were performed in triplicate. The relative expression was calculated based on the cycle threshold (CT) values of the target mRNAs normalized by the CT level of GAPDH [74] employing the well-established 2^-ΔΔCT^, and the data were analyzed using the Pfaffl method [63]. The sequence of the primers we utilized for the real-time PCR is listed in Table 1.

### 4.14. Protein–Protein Interaction Network Analysis

The PPI network analysis was prepared by employing the Search Tool for the Retrieval of Interacting Genes/Proteins (STRING) v. 10.5 databases (http://www.string-db.org), accessed on 30 May 2022 [75] to map the up-regulated and down-regulated target genes. The data for the protein interaction prediction were collected from different databases with the Kmeans clustering algorithm with 3 clusters based on a medium confidence score of 0.400.

### 4.15. DAPI Staining

For the investigation of nucleus morphology and chromatin condensation, DAPI staining was carried out. Both cell lines were cultivated in the 8-well chamber slides (5 × 10^3^ cells/well) and incubated for 24 h. Afterward, cells were treated with synthesized formulations and SUN. Subsequently, 24 h post-treating, cells were washed, fixed, and permeabilized using 0.1% (*W*/*V*) Triton X-100, stained with 200 μL/well of DAPI (200 ng/mL), and finally, PBS was added to the wells to keep the cells hydrated. The cells were investigated with Olympus IX81 inverted fluorescence microscope and an Olympus DP70 camera (Olympus Corporation, Tokyo, Japan).

### 4.16. Analysis of Oxidative Stress Induction

For evaluating the ROS generation in treated cells, the cell-permeable redox-sensitive dye DCFH-DA was used. Technically in this method, in the active cells, intracellular esterases hydrolyze DCFH-DA into DCFH, and the oxidation rate of DCFH into DCF displays the ROS level. Both cell lines were cultivated in the 8-well chamber slides (5 × 10^3^ cells/well) and treated with engineered NCs. After 48 h post-seeding, cells were washed and exposed to 5 μL DCFH-DA (10 mM) in a 500 μL medium and incubated for 30 min at 37 °C and the fluorescent intensity was detected using an Olympus IX81 inverted fluorescence microscope with an Olympus DP70 camera (Olympus Corporation, Tokyo, Japan).

### 4.17. Assessment of Mitochondrial Membrane Potential

Rhodamine 123 is a fluorescent lipophilic dye which can bind to the metabolically active mitochondria. Cells were cultured and treated with the same density and concentration as the previous assay. Finally, cells were washed and exposed to 5 μg/mL rhodamine 123 at 37 °C for 5 min and then washed with PBS and evaluated using an Olympus IX81 inverted fluorescence microscope with an Olympus DP70 camera (Olympus Corporation, Tokyo, Japan).

### 4.18. Statistical Analysis

The statistical differences between the control and test groups were conducted by ANOVA using GraphPad PRISM 8.0.1 software (GraphPad Software Inc., San Diego, CA, USA). Data were presented as mean ± standard deviation (SD). A *p*-value of less than 0.05 was considered statistically significant.

## 5. Conclusions

In the current investigation, MMSNP nanosystems were conjugated to MUC-1 aptamer and loaded with SUN to develop novel targeted delivery systems. The engineered MMSNP-SUN-MUC-1 with an average size of 97.6 nm and a zeta potential of −10 mv was characterized physicochemically. Further, the drug release study indicated a pH-dependent release. According to the flow cytometry assay, the MUC-1 aptamer armed NCs showed higher internalization and uptake in OVCAR-3 positive cells than the MDA-MB-231 negative cell line. Further, the engineered DDS appeared to be a successful nanosystem as confirmed by various in vitro cellular/molecular assays. While ROS production is induced in both cell lines in the case of naked MMSNP-SUN treatment, MUC-1 aptamer-armed MMSNP-SUN depicted low ROS generation in the negative cell line. As confirmed by the analysis of Bax/Bcl2 gene expression and annexin-V/FITC assay, apoptosis may be the main mechanism of cell death. In conclusion, it is envisioned that the developed MMSNP-SUN-MUC-1 can be considered an effective DDS, with minimum side effects and maximum therapeutic efficiency. However, the efficacy of the proposed system should be extensively investigated in the in vivo models of MUC-1-expressing ovarian cancer. Then, the efficiency of the fabricated nanosystem could be evaluated as a multifunctional agent exhibiting both therapeutic and diagnostic potential.

## Figures and Tables

**Table 1:** Primers used for real-time PCR assay.

mRNA	Forward Primer Sequence	Reverse Primer Sequence
Bax	5′-GATGCGTCCACCAAGAAG-3′	5′-AGTTGAAGTTGCCGTCAG-3′
Bcl2	5′-GTTCCCTTTCCTTCCATCC-3′	5′-TAGCCAGTCCAGAGGTGAG-3′
GAPDH	5′-CCTGCTTCACCACCTTCTTG-3′	5′-CCATCACCATCTTCCAGGAG-3′

## Data Availability

Not applicable.

## Sample Availability

Data will be available upon request.
